# Treatable traits identified in Chinese patients hospitalized with AECOPD: A Multicenter Cohort Study

**DOI:** 10.7150/ijms.111294

**Published:** 2025-04-13

**Authors:** Weiwei Meng, Jiankang Wu, Jiayu Wang, Rui Zhao, Sisi Liu, Naishu Xie, Qixuan Huang, Lijun Liu, Yanchao Liang, Huihui Zeng, Yiming Ma, Yan Chen

**Affiliations:** 1 Department of Pulmonary and Critical Care Medicine, The Second Xiangya Hospital, Central South University, Changsha, Hunan, China.; 2 Research Unit of Respiratory Disease, Central South University, Changsha, Hunan, China.; 3 Clinical Medical Research Center for Pulmonary and Critical Care Medicine in Hunan Province, Changsha, Hunan, China.; 4 Diagnosis and Treatment Center of Respiratory Disease in Hunan Province, Changsha, Hunan, China.; 5 Department of Pulmonary and Critical Care Medicine, People's Hospital of Shaodong, Shaoyang, Hunan, China.; 6 Department of Pulmonary and Critical Care Medicine, Zhuzhou Central Hospital, Zhuzhou, Hunan, China.; 7 Postdoctoral Station of The Second Xiangya Hospital, Central South University, Changsha, Hunan, China.

**Keywords:** chronic obstructive pulmonary disease, exacerbation, treatable traits, prognosis

## Abstract

**Background: “**Treatable traits (TTs)” is a precision medicine strategy for the management of chronic airway diseases. However, data on TTs in hospitalized AECOPD patients are limited. This study aimed to determine the prevalence of TTs in Chinese patients hospitalized with AECOPD and which traits predict future exacerbation risk, and to develop an exacerbation prediction model.

**Methods:** This multicenter, cohort study recruited patients hospitalized with AECOPD from January 2022 to April 2023. Participants underwent a multidimensional assessment to characterize the TTs and were then followed up for one year. Cox regression analyses were used to determine the association between TTs and future exacerbations and develop a prediction model.

**Results:** Finally, 28 TTs, including pulmonary (n=11), extra-pulmonary (n=12) and behavioral/risk factors (n=5) were identified. Five traits were associated with increased risk of future AECOPD readmission, including frequent exacerbations in the past year (adjusted HR: 2.079, 95% CI: 1.246-3.469), O_2_ desaturation (adjusted HR: 1.754, 95% CI: 1.001-3.075), eosinophilic airway inflammation (adjusted HR: 1.731, 95% CI: 1.078-2.777), pathogen colonization (adjusted HR: 1.852, 95% CI: 1.147-2.990) and gastroesophageal reflux (adjusted HR: 5.500, 95% CI: 1.923-15.730). Furthermore, one regression model was developed to predict personalized exacerbation risk and showed acceptable performance.

**Conclusion:** TTs can be systematically assessed in Chinese patients hospitalized with AECOPD, some of which are associated with future exacerbation-related readmission.

## Introduction

Acute exacerbation of chronic obstructive pulmonary disease (AECOPD) is characterized by worsening of respiratory symptoms, accompanied by a range of pathophysiological abnormalities such as exacerbated airway and systemic inflammation 1. The triggers of AECOPD are complex, often initiated by respiratory viral infections, which lead to and exacerbate bacterial infections in the lower respiratory tract, further worsening airflow obstruction 2. Patients with AECOPD exhibit significant heterogeneity in etiology, clinical presentations, phenotypes, comorbidities, and treatment responsiveness 3. A host of studies have confirmed that AECOPD is associated with a rapid decline of lung function, poorer health outcomes, increased hospital admissions and higher mortality 4-6. Therefore, systematically identifying the clinical characteristics of AECOPD patients and developing personalized intervention strategies holds substantial practical importance in improving patients' prognosis.

Treatable traits (TTs), first proposed by AGUSTI et al. 7, is an emerging field in precision medicine. It entails a multidimensional assessment of phenotypic or endotypic characteristics that are clinically relevant, identifiable and modifiable with treatment in patients with chronic airway diseases and can be specifically categorized into three domains: pulmonary traits, extrapulmonary traits, and behavioral/risk factors 8, 9. As a precision medicine strategy, TTs provide a structured framework for aligning therapeutic goals with personalized interventions. TT-based individualized medicine could enhance disease control, slow disease progression, improve quality of life and reduce healthcare utilization 10-13. Assessing and managing TTs in a holistic manner could be useful for patients with chronic airway diseases, particularly those with complex conditions such as hospitalized AECOPD patients 14, 15.

TTs have been explored in several studies in patients with asthma or chronic obstructive pulmonary disease (COPD)^16^, ^17^. However, there are scarce data from longitudinal studies investigating the prevalence of TTs among Chinese hospitalized patients with AECOPD and their relationships with future exacerbations. To address this knowledge gap, the aim of this study is to assess the prevalence of TTs in Chinese hospitalized AECOPD patients, and to identified TTs that can predict future exacerbation risk.

## Methods

### Study design and participants

This was a multicenter, observational cohort study conducted from January 2022 and April 2024. All Chinese subjects were from the COPD database set up by the Second Xiangya Hospital of Center South University that includes the Second Xiangya Hospital of Central South University, Zhuzhou Central Hospital, and People's hospital of Shaodong. In this study, hospitalized AECOPD patients at their first hospital admission were consecutively recruited between January 2022 and April 2023 and followed up for one year. The flow chart of patient enrollment is shown in Figure S1. The eligibility criteria included the following: (1) ≥18 years old; (2) inpatients with a primary diagnosis of AECOPD; (3) the presence of post-bronchodilator forced expiratory volume in 1 s (FEV_1_)/forced vital capacity (FVC) ratio<0.70 at baseline. Patients were excluded if they participated in other clinical trials or withdrew the informed consent.

### Sociodemographic information and clinical data collection

At admission, we collected the baseline characteristics including age; sex; body mass index (BMI); educational level; residence; smoking history; acute exacerbations (AEs) in the previous year, COPD history and comorbidities, modified Medical Research Council (mMRC) dyspnea grade, COPD Assessment Test (CAT) score, clinical COPD questionnaire (CCQ), inhalers and adherence. The inhalation technique was evaluated by clinicians. Data on other clinical investigations including blood tests (full-blood count, highly sensitive C-reactive protein (hs-CRP) and serum lipids), sputum culture, spirometry, Fractional exhaled Nitric Oxide (FeNO), 6-min walk test (6MWT), and high-resolution computed tomography/X-ray of the chest were also collected. All patients were followed up for one year. During the follow-up period, detailed information about exacerbation was collected through telephone interviews to assess patients' conditions, supplemented by a review of their electronic medical records for any hospital visits associated with acute exacerbations.

Exacerbations are defined as an acute worsening of respiratory symptoms that result in additional therapy. These events are classified as mild (treated with short-acting bronchodilators (SABDs) only), moderate (treated with SABDs plus antibiotics and/or oral corticosteroids) or severe (patient requires hospitalization or visits the emergency room) 18.

### Treatable traits identification

The TTs assessed in this study were based on published recommendations relevant to this concept 9, 16, 17. A total of 28 TTs were assessed, and the traits identified within the pulmonary (n=11), extrapulmonary (n=12), and behavioral/risk-factor domains (n=5) are presented in Table 1.

### Statistical analysis

Continuous variables are presented as the means with SDs when normally distributed or as the medians with IQRs otherwise. Categorical variables are expressed as counts (n) with percentages (%). Qualitative variables were compared using a χ^2^ test or Fisher's exact test, while quantitative variables were analyzed using Student's t-test, analysis of variance, or the Mann-Whitney U test. Correlations between the number of TTs and patients' health status (CCQ), CAT and FEV_1_% Pred were expressed using Spearman correlation coefficients. Cox proportional hazard regression model was used to determine TTs associated with AECOPD readmission within one year. Each trait was initially tested individually before we added those TTs that had *p*<0.10 from univariate analyses to the multivariate model. Meanwhile, visualized nomogram was drawn to facilitate the calculation of the predictive probability. Finally, the concordance index (C-index) the area under the receiver-operating characteristics curve with the calculation of the area under curve (AUC) replies was used to quantify the discrimination performance of the regression model 19. The Hosmer-Lemeshow goodness-of-fit test and calibration curves were used to assess the model calibration. A *p* value of more than 0.05 for the Hosmer-Lemeshow test suggests no evidence of poor goodness-of-fit, which is the desired outcome for a predictive model 20. All statistical analyses were performed with SPSS version 25.0 (SPSS Inc., Chicago, IL, USA) and R software (version 3.34). A *p* value of < 0.05 was considered significant.

## Results

### Baseline characteristics

Of the enrolled 560 participants, 84.8% (n=475) completed the one-year follow-up. Consequently, 475 participants were included for final analysis (Figure S1). The median age in the study population was 69.00 (IQR: 63.50-74.00) years. The median number of exacerbations in the previous year was 2.00 (IQR: 1.00-4.00) for all patients. The median FEV_1_% predicted was 33.00 (IQR: 23.90-43.00) (Table 2).

### Treatable trait prevalence

**Figure 1:**
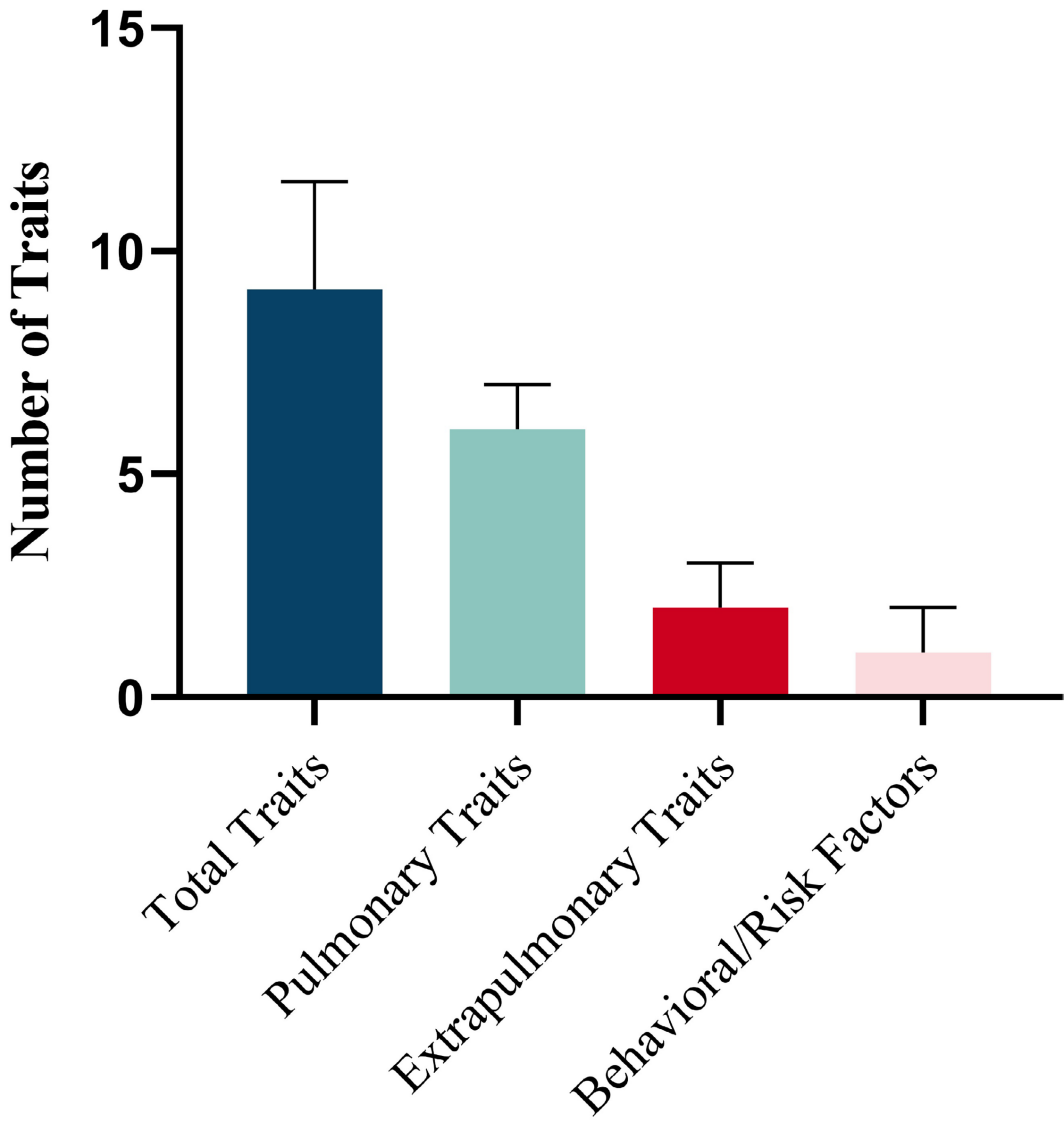

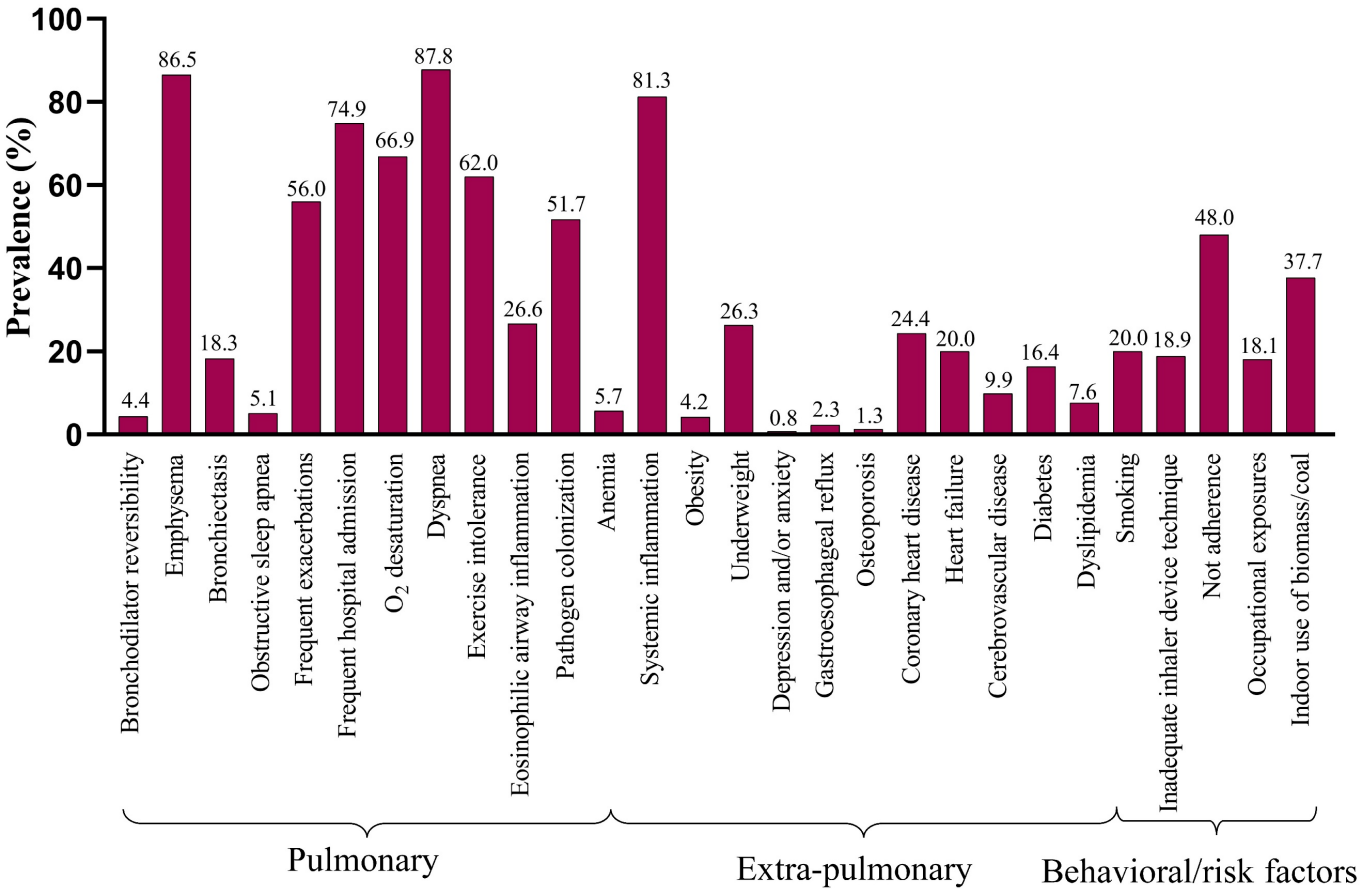
Prevalence of Treatable Traits. (A) Number of total treatable traits, pulmonary traits, extra pulmonary traits and behavioral/risk factors. Horizontal bars represent mean and standard deviation. (B) Prevalence of the investigated TTs in three domains.

The prevalence of individual traits is presented in Table 3. A mean (SD) of 9.11 (2.42) traits per patient were identified, comprising 5.68 (1.70) pulmonary, 2.00 (1.14) extrapulmonary and 1.43 (1.01) behavioral/risk-factor (Figure 1A). The prevalence of the 28 TTs evaluated in the study varied widely, from almost 90% (dyspnea) to < 10% in several other TTs (Figure 1B). In addition, the number of total traits was significantly associated with health status (CCQ) (r=0.293, *p*<0.001), and slightly associated with CAT (r=0.180, *p*=0.001) and FEV_1_% Pred (r=-0.146, *p*=0.0029) (Figure S2).

### Treatable traits and exacerbation risk

Among the 475 participants, 42.9% (n=204) experienced at least 1 moderate to severe exacerbation during the follow-up period, 17.3% (n=82) experienced at least 1 moderate exacerbation and 36.0% (n=171) experienced at least 1 severe exacerbation. The results found that, for each additional trait present, there was a 14.2% increase in the risk of future exacerbation-related readmission (HR:1.142, 95% CI: 1.056-1.237). And this risk will increase by 25.9% in the pulmonary domain (HR:1.259, 95% CI: 1.122-1.414, Table 4).

The relationships between TTs and future AECOPD readmission are presented in Table 4. The final prediction model's relationships among predictors were visualized using a nomogram. In the pulmonary domain, frequent exacerbations (adjusted HR: 2.079, 95% CI: 1.246-3.469), O_2_ desaturation (adjusted HR: 1.754, 95% CI: 1.001-3.075), eosinophilic airway inflammation (adjusted HR: 1.731, 95% CI: 1.078-2.777) and pathogen colonization (adjusted HR: 1.852, 95% CI: 1.147-2.990) were associated with increased risk of future severe exacerbations. In the extrapulmonary domain, the presence of gastroesophageal reflux (GERD) (adjusted HR: 5.500, 95% CI: 1.923-15.730) could increase the risk of future AECOPD readmission.

**Figure 2:**
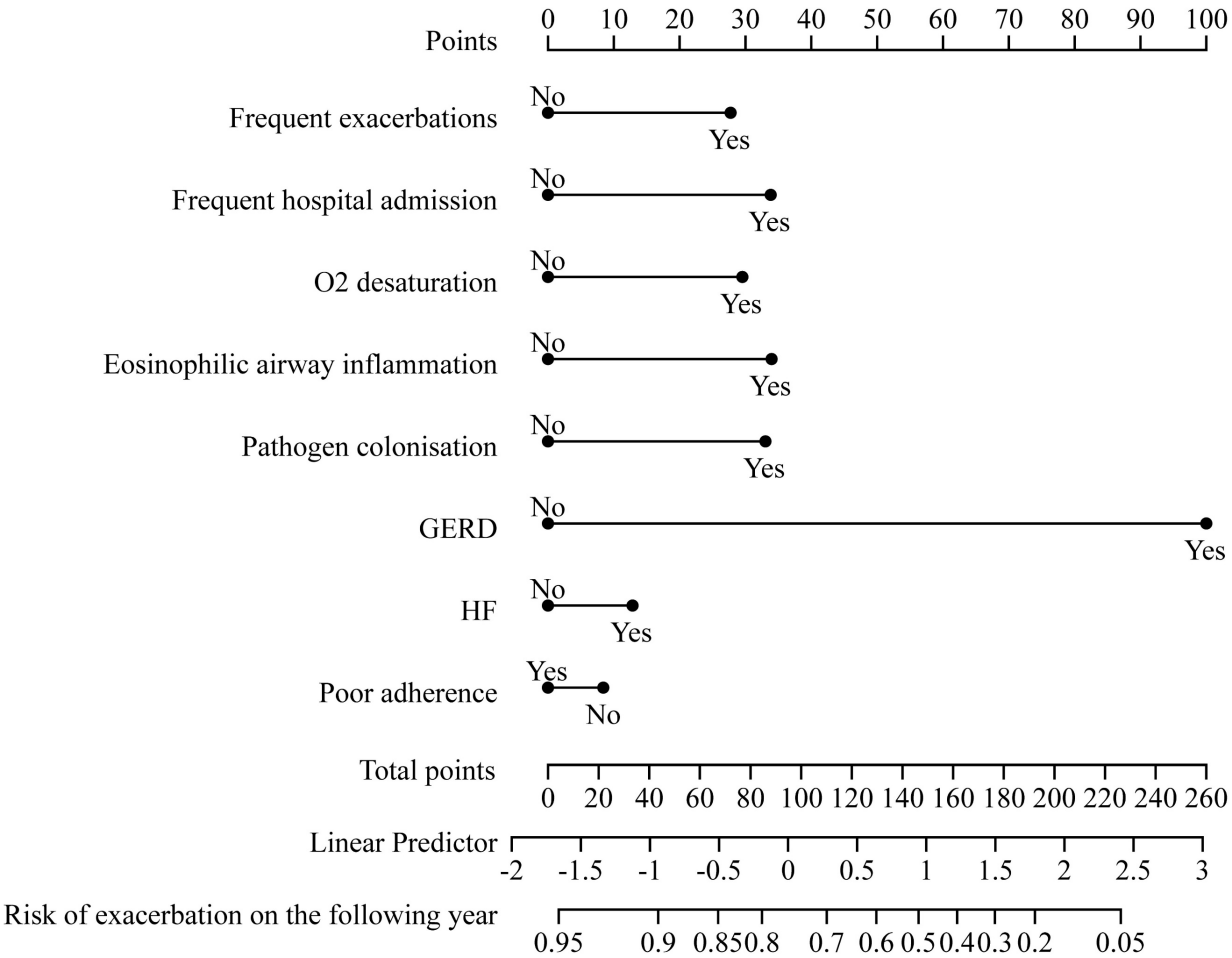
A nomogram predicting the risk of future exacerbation-related readmission for patients hospitalized with AECOPD. The value of each of variable was given a score on the point scale axis. A total score could be easily calculated by adding each single score and, by projecting the total score to the lower total point scale, we were able to estimate the probability of future exacerbation.

This cox regression model was visualized using a nomogram (Figure 2). It showed moderate discrimination in distinguishing between patients who did and did not experience AECOPD admission (C-index, 0.697, 95% CI, 0.633-0.761; AUC=0.720, 95% CI, 0.650-0.790, Figure S3). The goodness-of-fit of the model was evaluated using the Hosmer-Lemeshow test and calibration curves. The Hosmer-Lemeshow test yielded a nonsignificant statistic (*p*=0.597), which suggested that the model fit was acceptable. Evaluating the calibration curves for the prediction model of our study indicated good agreement.

## Discussion

This multicenter, prospective cohort study assessed the prevalence of numerous TTs in Chinese hospitalized patients with AECOPD and demonstrate the significant trait burden experienced by AECOPD patients. In addition, we identified five core traits that independently predicted future exacerbation-related hospitalization and developed a clinical prediction model that showed acceptable discrimination and calibration, allowing for personalized future AECOPD readmission risk prediction.

In this study, we assessed the prevalence of 28 potential TTs, including 11 pulmonary traits, 12 extrapulmonary, and 5 behavioral/risk traits. The high prevalence of TTs and the additional burden caused by them reinforce the need for multidimensional assessment and individualized precision treatment in hospitalized AECOPD patients. Furthermore, our study found that the number of TTs slightly positively correlated with the health status of AECOPD patients, indicating that we should not only evaluate the symptoms of patients, but also pay attention to the evaluation of patients' health-related quality of life in clinical practice. The weak correlation between the number of TTs and CCQ may be due to the wide variability of TTs prevalence among the three domains and the sample size.

COPD exacerbations have always been a vexing problem because of its close association with health outcomes 21. Its prevention is extremely important in the management of COPD 2. The correlations between the number of TTs and future exacerbation risk indicated that the precision medicine could be a pivotal strategy for improved outcomes for patients with AECOPD. We sought to determine whether TTs can be predictors of future exacerbations, which could provide key targets for future research. Cox regression analysis finally identified five predictive factors of future exacerbations including frequent exacerbations in the past year, exercise-induced O_2_ desaturation, eosinophilic airway inflammation, pathogen colonization and GERD. In concordance with several previous studies 22, 23, our results demonstrated that being prone to exacerbation in the past year could increase the risk of future exacerbation-related readmission. COPD patients with frequent exacerbations have a higher impairment of small airways 24, increased stable-state airway inflammation 25, and therefore greater disease severity, with worse clinical outcomes and prognosis 26, 27. Therefore, clinicians should pay more attention to the multidimensional assessment and individualized treatment of this subset of patients with frequent exacerbations.

Consistent with previous studies 28, 29, our study showed that eosinophilic patients have a greater risk of AECOPD readmission within one year after discharge, which further emphasized the clinical value of blood eosinophils in AECOPD. Several studies have reported the potential benefit of biological therapy in the subset of COPD patients with eosinophil-mediated airway inflammation 30, 31. A recent non-inferiority, multicenter RCT study of 93 AECOPD patients reported that blood eosinophil-guided glucocorticoid therapy at the time of an exacerbation of COPD was not inferior to standard therapy at reducing prednisolone use without affecting adverse outcomes 32. However, this study only focused on the treatment failure rate. Therefore, more prospective and RCT studies are needed to further demonstrate whether blood eosinophil-guided treatment can improve clinical outcomes and prognosis in this subset of AECOPD patients with eosinophilic airway inflammation.

The episodes of acute exacerbation (AECOPD) are mostly associated with colonized pathogens in the respiratory tract 33, 34. Evidence indicated that persistent pathogen colonization in the lower respiratory tract requires a lot of phenotypic adaptation and virulent mechanisms, such as host immune-inflammatory response, to cope with changing environmental pressures in the airway 35. The imbalance of respiratory microbiome, abnormal inflammatory response and an impaired airway immune system during acute exacerbation may provide an opportunistic platform for pathogen colonization resulting in a “vicious circle” 36. Multiple interactions between airway immune cells and colonized pathogens could lead to perpetuation of deleterious immune responses, accumulatively leading to further deterioration of COPD. Consequently, regular sputum culture test could be performed after discharge to monitor the lung microbiome for hospitalized AECOPD patients.

Prior studies have demonstrated that COPD is associated with GERD, and that GERD is associated with increased risk of future exacerbations among patients with stable COPD 37. Our results showed that GERD, as an extra-pulmonary trait, was the strongest predictor of future severe exacerbations in hospitalized AECOPD patients. Medical and surgical intervention are available for the treatment of GERD. Among medications, proton pump inhibitors (PPIs) are recommended as first-line therapy for people with GERD. Kang er al. 38 found that the risk of moderate exacerbation was significantly lower during the PPI treatment than at baseline in COPD patients. Therefore, it is recommended that healthcare professionals should diligently manage GERD present in AECOPD patients.

Using cox regression analysis, we developed a clinical prediction model to predict future AECOPD readmission. Our model had good calibration and predictive performance, demonstrating that it can predict future severe exacerbations. The nomogram can be conveniently used by clinicians.

Many studies consistently identify common predictors associated with future exacerbations in AECOPD patients, such as a history of prior exacerbations, eosinophilic inflammation, and comorbidities (e.g., GERD, heart failure) 39-42. Our findings align with these established predictors, further underscoring their critical role in clinical practice. Compared to previous studies, however, our study pioneers a comprehensive evaluation of AECOPD patients by integrating pulmonary, extrapulmonary, and behavioral/risk dimensions. This multidimensional approach enables our predictive model to adopt a highly individualized perspective, empowering clinicians to deliver personalized risk assessments and management strategies tailored to each patient's unique TTs.

There were some limitations in this study. Firstly, 28 TTs were selected based on previous literature and information available in our database, and thus not all COPD-related traits reported in the literature could be evaluated. Then, clinician or self-reported co-morbidities may have underestimated and overestimated prevalence. Thirdly, external validation of this prediction model is needed in the future research. Finally, we need to emphasize that this is a real-world study. RCTs designed to implement and test the TTs approach are complex but necessary. Prospective, longitudinal, interventional studies designed to explore whether precise treatment targeting TTs could improve management and prognosis for AECOPD patients are still urgently needed.

## Conclusions

TTs can be systematically assessed in Chinese hospitalized patients with AECOPD. Several TTs were identified as associated with future severe exacerbations including frequent exacerbations, O_2_ desaturation, eosinophilic airway inflammation, pathogen colonization, and gastroesophageal reflux. In addition, we presented a nomogram based on these specific TTs that can be conveniently used to allow personalized future exacerbation prediction in Chinese patients hospitalized with AECOPD. Future studies with individualized precision interventions targeting those core treatable traits should be conducted to evaluate their effects on health outcomes in AECOPD.

## Supplementary Material

Supplementary figures.

## Figures and Tables

**Table 1:** A full description of the definitions of TTs, including the assessment method and guide for identification

Treatable traits	Assessment Method	Guide for identification
Pulmonary traits		
Bronchodilator reversibility	Spirometry	FEV_1_ reversibility≥12%+≥200ml post-bronchodilator
Emphysema	X-ray/CT	Doctor and/or radiologist diagnosis
Bronchiectasis	Medical history or CT	Doctor and/or radiologist diagnosis
Obstructive sleep apnea	Medical history	Doctor diagnosis
Frequent exacerbations	Self-report	≥2 previous year
Frequent hospital admission	Self-report	≥1 previous year
O_2_ desaturation	6MWT	O_2_ desaturation levels of less than 90% during the 6MWT
Dyspnea	Questionnaire	mMRC≥2
Exercise intolerance	6MWT	6-minute walk distance < 350m
Eosinophilic airway inflammation	FBC; FeNO	Blood EOS≥0.3^*^10^^^9/l or FeNO≥30 ppb
Pathogen colonization	Sputum culture	Sputum culture positive for recognized bacterial pathogen
Extra pulmonary		
Anemia	FBC	Hb<110g/l for females, Hb< 120g/l for males
Systemic inflammation	FBC; hsCRP	hsCRP≥3mg/L or blood neutrophils≥6^*^10^^^9/l
Obesity	BMI	BMI≥30 kg/m^2^
Underweight	BMI	BMI<18.5 kg/m^2^
Depression and/or anxiety	Self-report/Medical review	Doctor diagnosis
Gastroesophageal reflux	Self-report/Medical review	Doctor diagnosis
Osteoporosis	Self-report/Medical review	Doctor diagnosis
Coronary heart disease	Self-report/Medical review	Doctor diagnosis
Heart failure	Self-report/Medical review	Doctor diagnosis
Cerebrovascular disease	Self-report/Medical review	Doctor diagnosis
Diabetes	Self-report/Medical review	Doctor diagnosis
Treatable traits	Assessment Method	Guide for identification
Dyslipidemia	TG; TC; HDL-C; LDL-C	TC≥5.2 mmol/l, LDL-C≥3.4 mmol/l, TG≥1.7mmol/l, HDL-C≤1.0 mmol/l
Behavior traits and risk factors		
Smoking	Self-report	Current smoker
Inadequate inhaler device technique	Questionnaire	Physician evaluation
Not adherence	Questionnaire	TAI≤46
Occupational exposures	Self-report	Medical history/Self-report
Indoor use of biomass/coal	Self-report	Medical history/Self-report

TTs: treatable traits; FEV_1_: post-bronchodilator forced expiratory volume in 1 s; CT: computed tomography; 6MWT: 6-minute walk test; mMRC: modified Medical Research Council dyspnea grade; FBC: full blood count; FeNO: Fractional exhaled Nitric Oxide; EOS: eosinophil; Hb: Hemoglobin; hsCRP: high sensitive C-reactive protein; BMI: body mass index; Hb: hemoglobin; TG: triglyceride; TC: total cholesterol; HDL-C: high-density lipoprotein cholesterol; LDL-C: low-density lipoprotein cholesterol; TAI: the test of adherence to inhalers.

**Table 2:** Demographic and clinical characteristics of all patients

Variables	Total (n=475)
Age (years)	69.00 (63.50, 74.00)
Sex, male	446 (93.9)
Body mass index (kg/m^2^)	21.05 (18.29, 23.77)
Educational level	
Primary	226 (47.6)
Secondary	119 (25.0)
High school	87 (18.3)
University and above	43 (9.1)
Smoking status	
Current smoker	95 (20.0)
Ex-smoker	328 (69.1)
Never smoker	52 (10.9)
Smoking index (pack-years)	40.00 (25.00, 60.00)
Years since diagnosis	6.00 (3.00, 10.00)
Exacerbations in the previous year	2.00 (1.00, 4.00)
Hospital admissions in the previous year	1.00 (1.00, 3.00)
CAT score	20.00 (16.00, 24.00)
mMRC dyspnea grade	3.00 (2.00, 4.00)
CCQ score	27.00 (16.00, 33.00)
Pre-admission inhalers	
No inhalers	77 (16.2)
LAMA	26 (5.5)
ICS+LABA	130 (27.3)
LABA+LAMA	57 (12.0)
ICS+LABA+LAMA	185 (39.0)
Post-BD FEV_1_% predicted	33.00 (23.90, 43.00)
Post-BD FEV_1_/FVC (%)	36.00 (30.00, 44.64)
FeNO	18.00 (12.00, 28.25)
CCI	1.00 (0.00, 2.00)

Data are presented as n (%) or medians (IQRs) unless otherwise stated. CAT: COPD Assessment Test; mMRC: modified Medical Research Council; CCQ: clinical COPD questionnaire; ICS: inhaled corticosteroids; LAMA: long-acting muscarinic receptor antagonist; LABA: long-acting beta-adrenoceptor agonist; Post-BD: postbronchodilator; FEV_1_: forced expiratory volume in 1 s; FVC: forced vital capacity; FeNO: Fractional exhaled Nitric Oxide; CCI: Charlson Comorbidity Index.

**Table 3:** Prevalence of TTs in the study participants with AECOPD

Treatable traits	AECOPD (n=475)	
	Expressed/Assessed^*^	%
**Pulmonary traits**		
Bronchodilator reversibility	21/475	4.4
Emphysema	411/475	86.5
Bronchiectasis	87/475	18.3
Obstructive sleep apnea	24/475	5.1
Frequent exacerbations	266/475	56.0
Frequent hospital admission	356/475	74.9
O_2_ desaturation	176/263	66.9
Dyspnea	417/475	87.8
Exercise intolerance	163/263	62.0
Eosinophilic airway inflammation	125/470	26.6
Pathogen colonization	244/472	51.7
**Extra pulmonary**		
Anemia	27/475	5.7
Systemic inflammation	386/475	81.3
Obesity	20/475	4.2
Underweight	125/475	26.3
Depression and/or anxiety	4/475	0.8
Gastroesophageal reflux	11/475	2.3
Osteoporosis	6/475	1.3
Coronary heart disease	116/475	24.4
Heart failure	95/475	20.0
Cerebrovascular disease	47/475	9.9
Diabetes	78/475	16.4
Dyslipidemia	36/475	7.6
**Behavior traits and risk factors**		
Smoking	95/475	20.0
Inadequate inhaler device technique	90/475	18.9
Not adherence	228/475	48.0
Occupational exposures	86/475	18.1
Indoor use of biomass/coal	179/475	37.7

TTs: treatable traits; AECOPD: acute exacerbations of chronic obstructive pulmonary disease. ^*^Expressed/Assessed represent the ratio of the number of people who expressed each TT to the number of people actually assessed in this study.

**Table 4:** Univariate and multivariate Cox regression analyses of TTs associated with AECOPD readmission within one year.

	Crude HR (95% CI)	*P*-value	Adjusted HR (95% CI)	*P*-value
**Total number of traits**	**1.142 (1.056-1.237)**	**<0.001**	-	-
**Pulmonary traits**				
Bronchodilator reversibility	0.674 (0.249-1.822)	0.437	-	-
Emphysema	1.083 (0.651-1.802)	0.759	-	-
Bronchiectasis	1.018 (0.659-1.574)	0.935	-	-
Obstructive sleep apnea	1.052 (0.491-2.252)	0.897	-	-
Frequent exacerbations	**1.659 (1.155-2.382)**	**0.006**	**2.079 (1.246-3.469)**	**0.005**
Frequent hospital admission	**1.791 (1.133-2.832)**	**0.013**	1.855 (0.939-3.666)	0.075
O_2_ desaturation	**1.971 (1.132-3.432)**	**0.017**	1.754 (1.001-3.075)	0.050
Dyspnea	1.522 (0.857-2.809)	0.147	-	-
Exercise intolerance	1.367 (0.839-2.228)	0.209	-	-
Eosinophilic airway inflammation	**1.559 (1.090-2.230)**	**0.015**	**1.731 (1.078-2.777)**	**0.023**
Pathogen colonisation	**1.751 (1.230-2.493)**	**0.002**	**1.852 (1.147-2.990)**	**0.012**
**Total number of pulmonary traits**	**1.259 (1.122-1.414)**	**<0.001**	-	-
**Extra pulmonary**				
Anemia	0.920 (0.430-1.969)	0.830	-	-
Systemic inflammation	1.394 (0.857-2.266)	0.181	-	-
Obesity	0.623 (0.230-1.686)	0.352	-	-
Underweight	0.819 (0.547-1.224)	0.330	-	-
Depression and/or anxiety	2.410 (0.596-9.743)	0.217	-	-
Gastroesophageal reflux	**2.867 (1.264-6.507)**	**0.012**	**5.500 (1.923-15.730)**	**<0.001**
Osteoporosis	0.589 (0.082-4.210)	0.598	-	-
Coronary heart disease	0.740 (0.482-1.136)	0.169	-	-
Heart failure	**1.597 (1.085-2.350)**	**0.018**	1.269 (0.724-2.224)	0.405
	Crude HR (95% CI)	*P*-value	Adjusted HR (95% CI)	*P*-value
Cerebrovascular disease	1.095 (0.629-1.905)	0.748	-	-
Diabetes	1.128 (0.719-1.768)	0.600	-	-
Dyslipidemia	0.897 (0.456-1.766)	0.754	-	-
**Total number of extra-pulmonary traits**	1.044 (0.888-1.228)	0.610	-	-
**Behavior traits and risk factors**				
Smoking	0.993 (0.647-1.525)	0.974	-	-
Inadequate inhaler device technique	1.339 (0.896-2.003)	0.155	-	-
Not adherence	**0.725 (0.514-1.024)**	**0.068**	0.855 (0.530-1.380)	0.522
Occupational exposures	0.981 (0.626-1.539)	0.934	-	-
Indoor use of biomass/coal	1.052 (0.741-1.494)	0.775	-	-
**Total number of behavioral/risk traits**	1.067 (0.888-1.282)	0.487	-	-

TTs: treatable traits; AECOPD: acute exacerbations of chronic obstructive pulmonary disease; HR: Hazard ratio.
