# Supplementary material for: Treatable traits identified in Chinese patients hospitalized with AECOPD: A Multicenter Cohort Study
Source: Int J Med Sci. 2025 Apr 13;22(9):2227–36. doi: 10.7150/ijms.111294 (PMC12035846; doi:10.7150/ijms.111294)
Supplement: Supplementary file 1 — Supplementary figures. [file ijmsv22p2227s1.pdf]

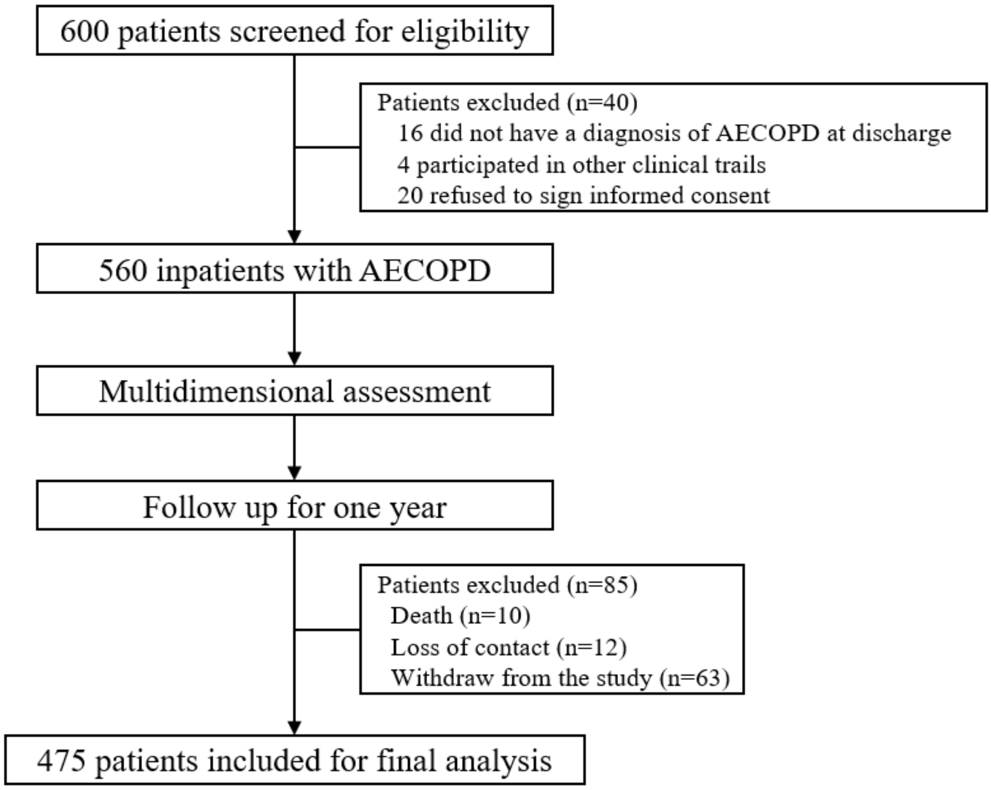

Figure S1. Flowchart of included patients. AECOPD: acute exacerbations of chronic obstructive pulmonary disease.

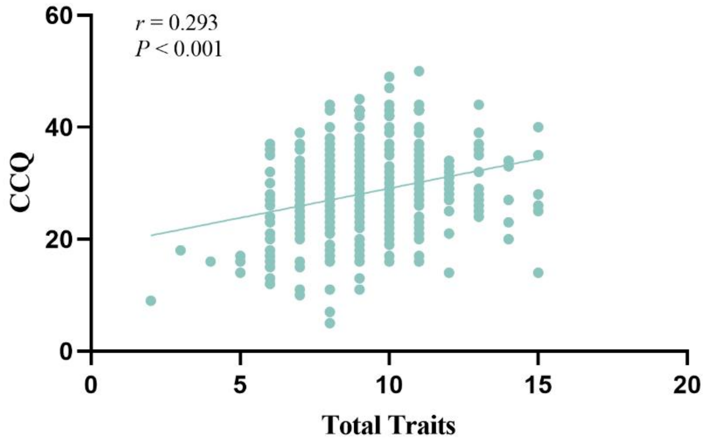

(A)

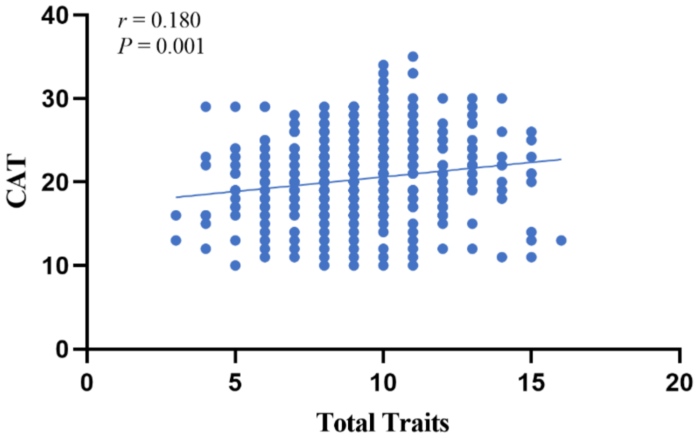

(B)

(C)

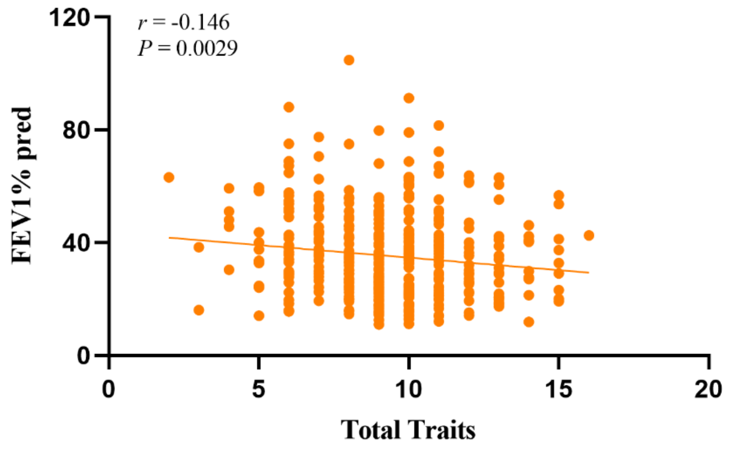

Figure S2. Correlation of total traits by health status (CCQ, A), CAT(B) and FEV_1% Pred(C) for inpatients with AECOPD. Diagonal line represents the linear regression line. CCQ: clinical COPD questionnaire; CAT: COPD Assessment Test; FEV_1: forced expiratory volume in 1 s.

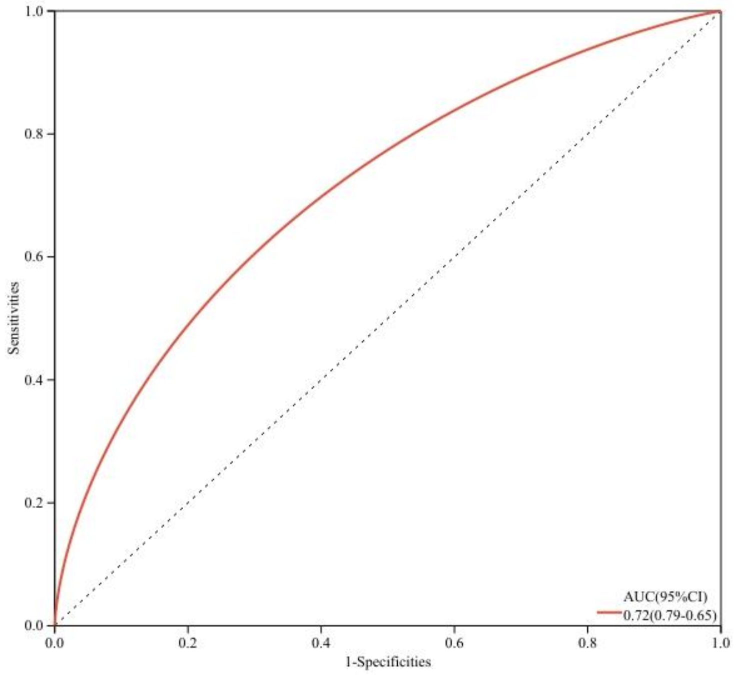

(A)

(B)

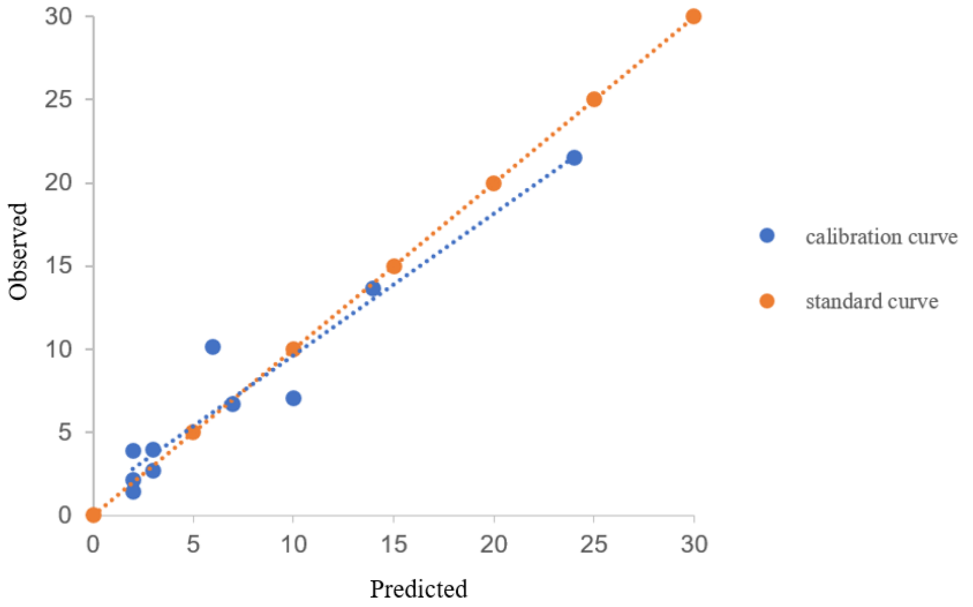

Figure S3. Efficacy of the Cox regression model. (A) Receiver-operating characteristic (ROC) curve of the prediction model. The x-axis specificity represents the true negative rate. The y-axis sensitivity represents the true positive rate. The AUC and the 95% CI are shown in the graph. (B) The calibration curves for the prediction model. Perfect prediction would correspond to the 45° orange dashed line. The blue dotted line represents the entire cohort (n=475), and the orange dotted line is standard curve (y=x), indicating observed nomogram performance.
